# Providing Continuity in Infant Mental Health Services for Medically Fragile Infants and Their Families

**DOI:** 10.1007/s10880-023-09957-1

**Published:** 2023-03-31

**Authors:** Patricia P. Lakatos, Naomi V. Rodas, Tamara Matic, Marian E. Williams, Laura L. Samora, Melissa C. Carson

**Affiliations:** Children’s Hospital Los Angeles, University of Southern California University Center for Excellence in Developmental Disabilities, 4650 Sunset Blvd, MS#53, Los Angeles, CA 90027 USA

**Keywords:** Infant mental health, Perinatal mental health, High risk infant follow-up, Trauma-informed care, Pediatric psychology

## Abstract

Having a baby who is prenatally or postnatally diagnosed with a medical condition places considerable stress on the parents, infants, and their developing relationship. Infant mental health (IMH) services offer an opportunity to address the challenges and support the parent-infant relationship. The present study outlined a continuum of care IMH program embedded within various medical settings of a large metropolitan children’s hospital. Applications of IMH principles within the fetal care center, neonatal intensive care unit, high risk infant follow-up clinic, and the patient’s home are described. Descriptive data about families served across settings and a case study are provided in order to illustrate the implementation of this unique IMH intervention model.

Having a child who is diagnosed with a medical condition or hospitalized can have a significant impact on the child, parent, and family. As such, pediatric hospitals have a variety of programs to support children and families, ranging from preventative services to ongoing behavioral health intervention. One model of intervention is an inpatient consultation liaison service staffed by pediatric psychologists, psychiatrists, and medical social workers that can be requested when children experience emotional or behavioral concerns during a hospitalization. Most pediatric consultation liaison services would be accustomed to receiving and treating a broad range of child and adolescent referrals but may be less accustomed to receiving referrals for infants. In one study of a consultation liaison service, only seven percent of consultations were for infants less than one year old; the majority of caregiver support requests in this age group were received from the intensive care units, including both the pediatric and neonatal intensive care units (NICU) (Piazza-Waggoner et al., 2013). In addition to inpatient consultation liaison services, there is a need for mental health consultation services in outpatient medical clinics where infants receive care. Some outpatient clinics who serve infants and their families have an embedded psychologist on their team who has expertise in infant development and developmental evaluation, given the specialization required in these content areas (Hoffman, et al. 2020). In order to address the unique challenges faced by infants with medical diagnoses and their families, there is a need for service delivery models that incorporate pediatric psychology (Palermo et al., 2014), adult mental health (Boyd et al., 2016), and infant mental health competencies (Weatherston et al., 2009).

Infant mental health (IMH) is defined as the “developing capacity of the child from birth to 5 years to form close and secure adult and peer relationships; experience, manage, and express a full range of emotions; and explore the environment and learn, all in the context of family, community and culture” (Zero to Three, 2019). IMH services seek to support the infant’s optimal emotional and developmental trajectory in the context of a nurturing relationship beginning in pregnancy and continuing through the early years of life. The goal is to prevent adverse outcomes for the young child and to intervene when biological, psychosocial, and environmental factors place the child’s development and attachment relationship at risk (Weatherston & Browne, 2016). Babies diagnosed prenatally or postnatally with a medical condition requiring hospitalization are at considerable risk for developmental (Caskey et al., 2014; Grunau, 2013; Johnson et al., 2015; Oudgenoeg-Paz et al., 2017; Rand & Lahav, 2014) and behavioral challenges (Lean et al., 2018; Vanderbilt & Gleason, 2010) due the disruption to co-regulatory opportunities that are the foundation for early development (Browne, 2021; Browne et al., 2016). They are also at risk for attachment disruptions given the stress associated with intrusive and discomforting medical procedures, separations often associated with a NICU stay, and parents’ reduced ability to buffer the infant from stress due to reduced proximity. In addition, parental stress and grief associated with worrying about their baby’s life, as well as challenges with required frequent medical care after discharge and concerns for the baby’s development can stress the infant-parent relationship. Furthermore, the birth of an infant can reactivate past histories of trauma and abuse, further risking transmission of challenges in parental care which can often be compounded by psychosocial risk factors such as poverty (Weatherston & Browne, 2016).

By utilizing an IMH lens when applying mental health consultation and liaison services in prenatal, perinatal, and postnatal clinical settings, assessment and intervention is broadened to encompass the earliest stages of development and the impact of the caregiving relationship in development. Studies have demonstrated the relationship between maternal–fetal bonding during pregnancy and post-partum bonding, as well as the role that post-partum depressive symptoms play in impacting attachment and bonding following birth (Dubber et al., 2015). Given the importance of assessing, identifying, and intervening for mental health needs during the prenatal and perinatal period, fetal care centers that offer fetal diagnosis, education, and treatment for pregnant people whose fetus has a health problem can also provide opportunities for the provision of mental health services that combine IMH and pediatric psychology or health psychology perspectives (Ashby et al., 2016; Dempsey et al., 2021).

Additionally, as consultation liaison services and models have developed, there has been a growing recognition of the need for mental health support within pediatric inpatient acute care units, such as NICUs. The role of psychologists within critical care units has been identified as an area of sub-specialty (Tunick, et al., 2013). Having an infant admitted to a NICU has been found to impact parental mental health through post-traumatic stress disorder, depression, and anxiety (Aftyka, et al., 2017; Alkozei, et al., 2014; Roque, et al., 2017). Practitioners have described the impact NICU hospitalizations have on the parent–child relationship and the close physical contact which is so important for the development of co-regulatory capacities (Browne & Talmi, 2017; Browne, et al., 2016; Weatherston & Browne, 2016). Therefore, the mental health consultant must be aware of how hospitalization in a NICU can impact the parent, the individual infant, and the parent-infant dyad (Hoffman, et al., 2020).

To provide a continuum of care, integrated IMH services should ideally begin in fetal care centers, continue in acute neonatal care settings, bridge to outpatient home-based services after hospital discharge, and continue in high-risk infant follow-up programs. Providing IMH services across these clinical settings increases access and decreases barriers to mental health services that may not otherwise be sought during this complex, overwhelming time. This continuity of IMH services also allows the trauma-informed, evidence-based intervention to be targeted and dose-specific to meet the changing needs of a family at each point in time.

The present study describes the implementation of a continuum of care IMH model at a metropolitan children’s hospital. Intervention approaches are described across settings including the fetal care center (FCC), neonatal intensive care unit (NICU), high-risk infant follow up program (HRIF), and the home. Descriptive data about families who received services across multiple settings in this continuum of care over a five-year period are presented. Finally, a case study will be presented to illustrate the IMH work provided across settings and highlight some of the benefits of implementing an IMH continuum of care model.

## Methods

### Development of the IMH Model Across Prenatal and Postnatal Settings

The IMH program described is affiliated with a well-established community mental health program within a children’s hospital in a metropolitan area. Leaders from the pre-existing program’s early childhood and pediatric psychology programs sought to expand existing services to meet the mental health needs of medically fragile infants and their families from the prenatal period through postnatal care. The IMH program was funded through a combination of public funds (a contract with the county mental health department to provide specialty mental health care to children with Medicaid insurance) and private foundation grants. This funding approach enabled the program to leverage philanthropic funds with insurance billing to make services accessible to as many families as possible. The FCC and HRIF services were entirely grant funded. Services in the NICU were funded either by grant funds or Medicaid billing, depending on the family’s insurance and identified needs for specialty mental health care. Extended outpatient and home visiting services were generally only available to families with Medicaid insurance; families with commercial insurance or no insurance had access to more limited home visiting support.

IMH services were provided by a total of four staff, including psychologists and marriage and family therapists who were trained and endorsed as infant and early childhood mental health specialists. Three of the four IMH specialists were bilingual, bicultural, Spanish-speaking clinicians. The primary work setting for these IMH specialists was the existing outpatient program where they had experience with medically complex young patients; they added roles in the other settings in order to meet the needs of infants and their families at earlier time points. Two of the IMH specialists were involved in at least two of the settings, FCC and NICU or NICU and HRIF, one was involved in FCC, NICU and HRIF, and all specialists had the option for home follow-up when needed. Reflective consultation, a space to explore therapeutic work with infants and families including the thoughts, feelings, and reactions evoked (Eggbeer et al., 2007), was offered monthly through group and individual approaches to support the providers in their IMH work.

Across the settings, a blending of consultation liaison models was necessary to meet the IMH consultation needs. In the NICU, a rotating team of IMH specialists accepted referrals for infants and families, similar to a traditional inpatient consultation-liaison model, with the exception that this team was dedicated solely to the NICU given the expertise needed in IMH. In the outpatient settings, embedded IMH providers were dedicated to that specific clinic and provided IMH consultation as part of the outpatient clinic appointment. IMH services provided in all settings were dose-specific ranging from single consultations to more intensive psychotherapy, based on setting, needs, or interest of the family.

At their core, infant mental health services are relationship-based, focusing on the needs of the baby, the parent, and their developing attachment relationship. Services are aimed at mitigating risk factors and supporting parental sense of competency in caring for a medically fragile child (Browne & Talmi, 2017; Weatherston & Browne, 2016). Within each setting, providers utilized a reflective stance and held a trauma- and diversity-informed lens rooted in Child-Parent Psychotherapy (CPP), an evidence-based dyadic intervention model for infants and young children who have experienced a traumatic event and/or disruptions in the attachment relationship and seeks to promote an optimal developmental trajectory (Lieberman & Van Horn, 2008; Lieberman et al., 2015). Infant mental health services seek to address parental distress and mental representations which can impact parental behavior based on how a parent understands the baby’s cues and communication. In addition, these services support parental competence in caring for a medically fragile baby utilizing developmental care principles to foster infant regulation, minimize distress, and strengthen the infant-parent relationship (Lakatos et al., 2019). Child-Parent Psychotherapy interventions include (a) supporting developmental progress, (b) providing unstructured reflective developmental guidance, (c) modeling appropriate protective behavior, (d) interpreting the feelings and actions of infants and parents, (e) providing emotional support and empathic communication, and (f) providing crisis intervention, case management, and concrete assistance with problems of living (Lieberman & Van Horn, 2008; Lieberman et al., 2015). These interventions are individualized for each setting along the continuum of care.

IMH services require understanding and holding the perspective of the multiple providers involved in the care of a medically fragile child prenatally and postnatally. These might include neonatologists, perinatologists, nurses, medical social workers, and other medical subspecialties as well as early intervention providers. All services are provided in collaboration with the medical team and the medical social workers, with regular discussions about the roles of various team members to ensure good coordination. In addition, the IMH specialists utilize a reflective stance to support team members in expanding their understanding of infants and families and offer insight and strategies to address the strong reactions the work with families in these intensive settings may bring (Browne, 2021; Browne & Talmi, 2017; Browne et al, 2016). In addition, the IMH team supports staff in fostering the infant-parent bond and parental sense of competence in caring for their medically complex baby.

### IMH Intervention Settings and Role of IMH Specialist

Table 1 provides an overview of the settings described below, including the focus of medical care, the interdisciplinary team, the family experience, unique roles of the IMH specialist in that setting, and the number of IMH sessions (average and range) provided per family.

**Table 1:** Overview of infant mental health services across the continuum of care

Infant mental health (IMH) principles implemented by IMH specialists across all settings					
• Relationship-based approach that supports parent-infant attachment and parental competency					
• Support for infant’s emotional and developmental trajectory; goal to prevent adverse outcomes					
• Trauma-informed care: addressing pediatric medical traumatic stress, parental mental health, and other psychosocial stressors					
• Child-Parent Psychotherapy informed interventions					
• Diversity-informed approach that reduces barriers to accessing care					
• Reflective practice applied to work with families and staff					

Unique aspects of each setting					
Setting	Focus of medical care	Interdisciplinary team	Family experience	Unique roles of infant mental health specialist	Number of IMH sessions: mean/median (range)
Fetal care center	High-risk, medically complex pregnancies	Perinatologist, multiple physician subspecialists, nurse care managers, IMH specialist	Prenatal medical outpatient visits lasting 1 to 3 h and occurring about once per month, beginning when family learns of fetal abnormality and increasing to weekly visits close to delivery	Embedded in medical outpatient clinic Time-limited trauma-informed consultation Help parents manage distress, prepare, and support fetal attachment Support parent during ultrasound or specialist visits and during visits to NICU Support medical team to understand parent’s psychological experience	1.02 (1–7)
Neonatal intensive care unit	Survival of infants with complex medical conditions	Neonatologists, medical subspecialties, nurses, social workers, rehabilitation professionals (lactation, occupational therapy), respiratory, IMH specialist	Inpatient intensive care hospital stays ranging from 2 weeks to one year (average 2–4 months); most infant care provided by medical providers; parents may be separated from infant for long periods	Bedside sessions with family Explore pregnancy, birth and NICU hospitalization experiences; support cohesive narrative Address parental distress and mental health needs impacting attachment Interventions at bedside to support parents’ attunement to baby’s cues, competence in caring for baby, and bonding Support parents in preparing for transition home	6.05 (1–20)
High-risk infant follow up clinic	Monitor medical and developmental needs and link to early intervention and community supports	Developmental behavioral pediatricians, nurse manager, dietitian, occupational and physical therapist, social worker, IMH specialist	Four outpatient visits in first 3 years of life; visits include multiple team members	Embedded in interdisciplinary clinic Process feelings regarding transition home from hospital and experience of parenting a medically fragile baby Support parents’ growing understanding and acceptance of their child’s developmental delays and differences Identify behavioral or social-emotional concerns in the child Link to IMH and/or adult outpatient services or parent support groups when needed	1.50 (1–3)
Infant mental health home visiting	Outpatient monitoring and treatment of long-term challenges associated with medical needs	IMH specialist; consultation with medical providers, rehabilitation professionals, early intervention providers	Adjusting to caring for infant at home; frequent medical and early intervention appointments	Amount of service varies depending on need, parent preference, location of home, and funding source available Continuous assessment of changing needs and developing parent-infant attachment Help parents navigate system of care Provide full Child-Parent Psychotherapy model when indicated Support needs of other family members (e.g. siblings, grandparents)	Med = 3.00 (1–125)

#### Fetal Care Center

The Fetal Care Center (FCC) is an integrated multidisciplinary program, led by a perinatologist with the support of nurse care managers. It integrates more than twenty pediatric sub-specialties including pediatric surgery, fetal cardiology, and neonatology. The goal of the program is to support pregnant people and their families who are preparing for the birth of a baby that has been identified prenatally as having a complex medical condition, developmental abnormalities, risk of premature birth, or birth complications that require integrated care prenatally and post-birth including neonatal intensive care services. The model for care offers the continuum from fetal diagnosis through timed delivery in a single program for the most at-risk pregnancies, therefore minimizing fragmentation of care and conflicting information for families.

The clinic serves over 500 pregnant people annually from a large metropolitan area and across counties and provides a variety of services including prenatal diagnosis and interventions, comprehensive case management which involves perinatal and delivery management and coordination, as well as integrated IMH services (Ashby et al., 2016). IMH specialists are embedded in the program on each clinic day to provide time-limited trauma-informed consultation and support to pregnant people and their immediate family. The goal is to support them in managing the distress that is often associated with fetal diagnoses, minimize disruptions to the developing attachment relationship with the baby, optimize coping skills, and help parents prepare for what lies ahead. Consultations occur during the medical appointment when difficulty coping with a diagnosis is identified, with some patients receiving up to four contacts, and others one or none, depending on a person’s interest. Nurse care managers most often refer the following patients: families who will have a baby admitted to the NICU following birth; teen pregnancies; and families who have a history of mental health concerns or extensive psychosocial needs.

IMH support includes opportunities for the family to process information received during appointments and address grief or trauma associated with it, being present during the ultrasound or specialist visits to help the parent verbalize and address concerns, providing emotional support or a space to explore decisions regarding the pregnancy, or simply bearing witness and holding the depth of the experience. Services include linkage to adult outpatient mental health services when warranted. Twenty five percent of babies followed prenatally by the FCC are transferred to the NICU at the pediatric hospital in this program once stabilized at a community hospital where they are born. This often requires that the baby and mother be separated for several days until she is discharged from the birthing hospital. Therefore, the IMH specialist helps the parents prepare by taking them to visit the NICU and connecting them with the social work and IMH team in that setting to facilitate a smooth transition.

Support for the FCC medical team includes consultation prior to the visit and debriefing after the appointment to foster greater understanding of the expectant parent’s psychological experience. Together, the IMH specialist and the medical team help the parent(s) anticipate the birth of the medically fragile baby and what might be involved during the delivery and hospitalization. The IMH provider seeks to foster fetal attachment and address challenges in the transition to parenting a medically impacted infant.

#### NICU

The level IV NICU has approximately 600 annual admissions of critically ill infants who require neonatal medical and surgical subspecialty services, including extracorporeal membrane oxygenation (ECMO) and total body cooling. The unit has single and two-family rooms to promote increased parental presence and participation in the routine care of babies. Infants often require long term hospitalizations of up to a year with most staying between two and four months. Once hospitalized, social workers conduct psychosocial assessments with all families and identify those needing services from the IMH specialists in the NICU. Additional information about the decision-making process and pathways regarding referrals to the IMH service are outlined in Driver et al. (2021). In cases where the family received services in the FCC prior to the baby’s birth, the IMH specialist who worked with them in the FCC continues to work with them in the NICU. Following is a summary of these services with greater detail provided by  Lakatos et al. (2019).

IMH services in the NICU begin with an initial assessment phase that focuses primarily on building a therapeutic relationship and helping the parent feel a sense of safety within the crisis-driven medical environment. The IMH specialist explores pregnancy and birth experiences as well as past involvement with the medical system to build rapport, normalizes the often-present traumatic response to the medical milieu, fosters parental regulation, and supports the parents in creating a cohesive narrative of their experience. During this initial phase, the IMH specialist assesses distress or mental health needs in the parents, siblings, or extended family members. The IMH specialist explores psychosocial stressors or past traumatic experiences which may impact parent-infant attachment. As part of this process, the IMH specialist helps parents begin to grieve the loss of the healthy imagined baby and reconsider their roles within the context of the baby’s new medical reality. The IMH specialist assesses parents’ coping capacities and listens for how they are making meaning of the experience. In doing so, the IMH specialist notices areas of acceptance and resilience as well as possible vulnerabilities that may require additional exploration and can begin to determine the level of intervention needed. For families with good coping skills and a strong bond with their baby, a brief intervention might be all that is needed; for others where there are greater risk factors and concerns regarding the impact on the parent-infant bond, a more intensive intervention may be warranted. This CPP-informed intervention focuses on supporting the parent-infant bond by fostering the parents’ attunement to the baby’s cues, which can often be harder to read in the medically fragile baby and threaten the parents’ sense of competence and connection to the baby. The IMH specialist helps the parent support the baby’s regulation while parent engages in the baby’s care as well as hold the baby or provide kangaroo care when medically feasible. As the baby gets ready to be discharged from the hospital, the IMH specialist provides a space to reflect on the intense and complex emotions associated with the transition home and prepare for the change in their role with the baby.

#### High-Risk Infant Follow-Up Clinic

The HRIF clinic provides developmental monitoring and linkage to services for infants with complex medical and developmental needs after hospitalization in the NICU. Infants are eligible for follow-up due to low birth weight (less than 1500 g), premature birth (gestation less than 32 weeks), or a variety of neurologic and/or cardiovascular issues that place brain development at high risk. Infants that meet these criteria are referred to HRIF by the Clinical Care Coordinator through an electronic system during preparation for discharge from the NICU and the caregivers receive the referral information on their discharge summary. This multidisciplinary setting includes a nurse care coordinator, pediatrician or developmental behavioral pediatrician, dietician, physical and occupational therapists, social worker, and IMH specialist. Notably, the IMH specialist or psychologist is not a required discipline in HRIF clinics in the state where this metropolitan hospital is located (Department of Health Care Services, 2022); however, clinic leadership determined this discipline to be a valuable addition to address the behavioral and mental health needs of infants and families after NICU hospitalization. Infants are seen soon after they are discharged from the hospital and at approximately 6 months, 12 months, and 24 months of age corrected for prematurity, with the possibility of having one extra visit if there is need for further follow-up or to ensure linkage to services.

In all appointments, the IMH specialist provides a space in the busy clinic setting where parents can share and process their experiences adjusting to their baby’s evolving needs. This includes normalizing the parents’ experience and feelings and reflecting on their strengths to build their parenting confidence. Sometimes this brief intervention during the consultation is enough to help families move forward. Others may need more support, especially if there are concerns for the parent-infant relationship, including difficulties recognizing and responding sensitively to the infant’s cues or challenges in effective co-regulation. Then linkage is made to outpatient mental health services or other community-based supports, e.g., parent support group, and/or adult mental health services.

The IMH specialist recognizes the critical role the parent-infant relationship has on neurodevelopmental outcomes and the unique opportunity to intervene during particular “touchpoints” (Brazelton & Sparrow, 2003). These are times when parents may be feeling especially vulnerable because their infant is moving into a new developmental phase that results in disorganization of the family system, or parents expect a new milestone that is not happening because of the infant’s delays. For example, the first appointment typically occurs shortly after the baby has been discharged from the hospital. Now that the life-threatening crisis has stabilized, parents may find themselves flooded by thoughts and feelings they held at bay during the hospitalization. This timing provides a window of opportunity for the IMH specialist to support the family’s adjustment to the transition home, process feelings of stress and worry, reinforce coping strategies, and address concerns about feeding and sleep.

The 6- and 12-month appointments are times when many parents begin to recognize their infant’s developmental delays and differences. The IMH specialist then has opportunities to address the family’s worries about the future, feelings of overwhelm associated with medical appointments and special services, or their struggles with barriers in accessing services. The 24-month appointment serves as a good checkpoint to assess behavioral or social-emotional concerns and provide therapeutic consultation regarding how to obtain further assessment, e.g., an evaluation for autism spectrum disorder and/or developmental services. Additionally, information and therapeutic guidance about vulnerable child syndrome (Green & Solnit, 1964) may be warranted for some families during this stage when toddlers are expected to need increased autonomy to support their development.

#### Home Visiting

Home visiting services are typically planned prior to discharge from the hospital and decided through a collaborative, family-centered discussion with the parents as part of the discharge planning process. All families involved with inpatient IMH services were eligible for and were offered one to two home visits; exceptions were those families who lived far from the hospital, who were offered telephone support. Families whose child had Medicaid insurance were eligible for longer-term home visiting services through the agency’s contract with the County Department of Mental Health, and provision of those services was determined based on family preference and clinical judgement about the appropriateness of longer-term mental health services. Many parents feel it would be helpful to continue receiving therapeutic services during the often anxiety-provoking transition home. Other parents may be eager to leave the NICU behind, and all that is associated with it, and may choose not to continue therapy services. IMH services after discharge are tailored to a family’s needs and vary from one or more phone calls or home visits to weekly therapy in the family’s home. For some families, the infant’s medical crisis is the only major stressor, and a short-term approach is sufficient to help them adjust and rebound from the stress. However, for many families seen in this urban medical setting, the infant’s medical crisis is an additional major life stressor among others, which has a cumulative effect.

The IMH specialist is continuously assessing and reassessing the family’s adjustment to the ever-changing demands of caring for an infant with special medical, and often developmental needs to help determine the specific dose of services that is required. During phone sessions and home visits, the IMH specialist continues to use a CPP-informed approach. This includes assisting parents in navigating the various systems of care and linking with the necessary specialty medical clinics and early intervention services in the community. These tasks can feel daunting to exhausted parents that are transitioning home from the hospital. Additionally, the IMH specialist may decide that the full CPP intervention is necessary, including a more thorough assessment of the parents’ history of stress and trauma, that will be used to inform the intervention plan. In any case, the IMH specialist continues with the interventions that likely began during the hospitalization, including validating the parent’s feelings and experiences, processing the stress or trauma of the birth and hospitalization and making new meaning of the experience, building parents’ effectiveness and confidence in reading and responding to their infant’s cues and providing co-regulation, and consistently supporting the parent-infant relationship as they move from the role of medical caregiver to parent. The latter involves supporting parents in getting to know their baby and finding the joy in parenting. Additionally, the IMH specialist may help other family members, such as older siblings or grandparents, adjust to the infant being home, and may help to address communication and co-parenting challenges.

### Study Design and Participants

The study was approved by the Institutional Review Board at the host institution. A retrospective chart review was conducted, covering the period from January 2014 through May 2019. The chart review included demographic information (infant’s race/ethnicity, gender of infant, and preferred language of parent), insurance status (Medicaid or commercial insurance), and information about the services provided (location of services and number of sessions). This study included a subsample of a study of IMH services in the NICU setting that examined referral pathways to the IMH program (Driver et al., 2021). Finally, a family was selected for the case study that was representative of the families served, illustrated applications of the continuum of services across settings, and consented to have their story shared in this manuscript.

A total of 311 families in the NICU were referred for IMH services in the time period of study, and 194 (62%) referred families received IMH services; the remainder declined services or could not be reached by the IMH clinician. Additional information about the referral pathways and factors that were associated with engagement in IMH services is provided in Driver et al. (2021). This study, focused on the continuum of care across settings, analyzed data from the 89 families who had received IMH services in the NICU and at least one of the other settings described above (see Table 2); this sample represented 46% of the total number of families that received any IMH service in the NICU. The sample of infants was 47% male. Regarding their racial/ethnic identities, the sample represented more Hispanic/Latinx and Black/African American individuals and fewer Asian and White non-Hispanic individuals than the hospital’s total NICU population. In the study sample, 66.3% identified as Hispanic/Latinx (versus 53% in NICU), 11.2% identified as Black/African American (versus 9.5% in NICU), 7.9% identified as White non-Hispanic (versus 21.1% in NICU), 3.4% identified as Asian (versus 8.0% in NICU), and 11.2% did not have this information in their medical record (versus 6.9% in NICU). English was identified as the preferred language by 74.2% of parents and Spanish was identified as the preferred language by 24.7% of parents.

**Table 2:** Clients served in multiple IMH locations (N = 89)

Number of IMH settings	*n*	%
Two	58	65.2
Three	28	31.5
Four	3	3.4

### Data Analysis

Chart review data was entered into SPSS and descriptive statistics were computed. Group comparisons were calculated using *t*-tests (continuous variables) and Chi-square tests (dichotomous variables) regarding the treatment variables, comparing groups by language (English or Spanish) and insurance status (Medicaid or commercial).

## Results

Due to the inclusion criteria, all families had received IMH services in the NICU setting. In addition to services within the NICU setting, 22 (24.7%) families engaged in IMH services in the FCC, 53 (59.6%) families engaged in IMH services through the HRIF, and 48 (53.9%) families engaged in IMH services provided in their home. Table 2 provides information about the number of families participating by number of settings. On average, families engaged in 16 IMH sessions across multiple settings. We examined the length of IMH treatment in quantiles and found that 23 (25.8%) families engaged in 1–4 sessions, 26 (29.2%) families engaged in 5–10 sessions, 18 (20.2%) families engaged in 11–17 sessions, and 22 families (24.7%) engaged in 18 or more sessions. Families with Medicaid insurance engaged in significantly more IMH service sessions than families with commercial insurance (Table 3). Spanish-speaking parents were significantly more likely to have been seen in their home compared to English speaking parents. English speaking parents were, on the other hand, more likely to have engaged in IMH services within the HRIF Clinic (Table 3). No differences by language or insurance status were seen in the engagement in services in the FCC or NICU.

**Table 3:** Treatment characteristics by insurance type and primary language

	Insurance type			Primary language of parent		
	Medicaid insurance (*n* = 52)	Private insurance (*n* = 37)	*t* or *x*^*2*^	English (*n* = 66)	Spanish (*n* = 22)	*t* or *x*^*2*^
Number of IMH treatment settings	2.40 (.53)	2.40 (.56)	*t* = -0.43	2.35 (.48)	2.45 (.73)	*t* = -0.63
Number of total sessions	21.31(27.64)	9.00 (5.78)	*t* = -3.11**	14.88 (23.69)	20.27 (17.55)	*t* = -0.98
Percentage treated at Fetal Care Center	21.1%	29.7%	*x*^*2*^ = 0.85	21.2%	36.3%	*x*^*2*^ = 2.02
Percentage treated at home	61.5%	43.2%	*x*^*2*^ = 2.91	46.9%	72.7%	*x*^*2*^ = 4.39*
Percentage treated at high-risk infant follow-up program	57.6%	62.1%	*x*^*2*^ = 0.17	66.6%	36.3%	*x*^*2*^ = 6.26*

*p < .05, ** p < .01

### Case Study

The following is a case example where IMH services were initiated in the FCC, and then continued in the NICU and home with the same IMH provider in all three settings. The case will illustrate the IMH CPP-informed services provided. The family gave consent to share their story for this article; in order to protect the family’s confidentiality, the names and some other demographic details were changed.

Sofia was referred by her obstetrician to the FCC following her first ultrasound with the perinatologist when abnormalities in the fetus’ kidneys were identified. Sofia came to the clinic accompanied by her mother for the first visit. Marcos, the baby’s father, joined during subsequent visits. Sofia was a 16-year-old high school student, born in the U.S. to Spanish monolingual undocumented immigrants from a small town in Mexico. She lived in a one-bedroom apartment with her parents, her paternal grandmother, and three younger siblings including an infant. Sofia met Marcos online and they barely knew each other before they became pregnant. Marcos was 15 years old, born in the U.S. and had some learning challenges. He lived with his parents, both documented immigrants from Mexico.

During Sofia’s first ultrasound at the FCC, the physician identified one multicystic kidney and could not visualize the other. This was later confirmed via magnetic resonance imaging (MRI). As her pregnancy progressed, Sofia presented with low levels of amniotic fluid and concern for pulmonary hypoplasia.

Sofia was introduced to the IMH specialist by the nurse care manager during her first visit. She was noticeably quiet, did not make much eye contact, responded with short phrases when any of the providers tried to explore her thoughts and feelings, and would turn to her mother and ask her what she should do. Sofia’s mother encouraged her to express her thoughts but questioned whether Sofia should continue the pregnancy, worrying about the impact that caring for a medically fragile baby would have on her life. Marcos’ mother attended one of the initial appointments and shared similar views to Sofia’s mother. Despite parental concerns, Sofia was clear she wanted to continue the pregnancy, while acknowledging her own fears.

Sofia was initially seen once a month at the FCC and as the pregnancy progressed, she was seen every 2 weeks and then weekly. The IMH specialist saw her for a total of seven visits until she delivered her baby. Her mother was always present; Sofia would become anxious, shake, and bite her nails if her mother had to step out momentarily for any reason. Marcos came for most of the appointments with support from Sofia’s mother who would drive them both. The IMH specialist was there for every visit. She was present in the exam room during Sofia’s ultrasounds, in meetings with specialists to facilitate their interaction and help the family ask any questions and had opportunities to meet alone with the family to offer support. The IMH specialist took the family on a tour of the NICU during the pregnancy, to help orient them to this setting and offer additional opportunities to address concerns. The nurse care manager expressed relief knowing that the IMH specialist would be supporting her patient and often talked about her worries regarding this family, expressing gratitude for how the IMH specialist supported the medical team with Sofia’s care.

Both parents seemed intimidated by the whole medical experience but managed it somewhat differently. Throughout her follow up at the FCC, Sofia was very stoic. She appeared to understand the information offered and limited her brief questions to concrete aspects of the baby’s care such as asking if the baby would be cold, but rarely inquired about the baby’s health or much needed medical treatment. Marcos did not ask any questions but was more emotional during the visits. Sofia’s mother felt comfortable sharing her concerns and obtaining the information she needed to help Sofia, but often wondered what it would be like to have a medically fragile baby in the home. The IMH specialist offered support to all of them and felt she was a steady presence for these parents, often standing or sitting next to them and serving the role of a therapeutic companion. At times, given Sofia’s quiet engagement, the IMH specialist would wonder about her interest in services and Sofia would reiterate her desire for support and state she wanted the IMH specialist to come and see her once the baby was in the NICU.

After baby Maria was born and transferred to the NICU, Sofia called the IMH specialist and asked her when she was coming. Maria was in the NICU for 2 weeks and the IMH specialist visited the family several times in the hospital. As part of these visits, the IMH specialist explored Sofia’s experience with the delivery and initial separation from her baby and then focused on supporting the parent-infant relationship. The IMH specialist encouraged Sofia and Marcos to hold and talk to their baby and helped them notice Maria’s cues. She also supported Marcos and Sofia in conversations with the medical team to help them understand any signs of kidney failure and Maria’s eventual need for dialysis and kidney transplant.

After Maria was discharged from the hospital, the IMH specialist continued providing services in the home until Maria was 3 years old and transitioned out of early intervention services. The IMH specialist worked with Maria and Sofia as well as Sofia’s extended family. She also worked with Marcos when he visited the baby during one of their appointments or at the hospital. Their appointments were inconsistent given the number of times Maria was sick or had other appointments. The IMH specialist sought more consistency by accompanying the family during medical appointments or during many of Maria’s hospitalizations. The IMH specialist also supported Maria’s linkage with early intervention services and helped Maria’s parents implement strategies recommended during those visits. The IMH specialist was a strong link to the various systems of care as she helped the family become more comfortable navigating these systems on behalf of Maria and to address the educational needs of Sofia’s siblings.

Maria was a happy, easy-going, and friendly baby who was curious about her environment and progressed well developmentally, with some setbacks following her hospitalizations. She also had periods of extreme fussiness associated with swelling related to kidney failure. Everyone in the family helped care for Maria and they were all mostly present during home visits. Sofia diligently took care of all of Maria’s needs but struggled with her ability to offer empathy when Maria was in pain; the IMH specialist offered support in this area. She noticed, for example, how Sofia followed the daily injection protocol established by the medical team. During these procedures, the IMH specialist would intervene on behalf of the parent-infant dyad by speaking for the baby to share how well her mother cared for her, but also letting Sofia know that the shots were painful. The IMH specialist helped Sofia prepare the baby for these daily shots by using narration and modeled empathy for the baby. The IMH specialist noticed that Sofia was very quiet with the baby and worried she was not engaging with her baby and deferring care to others. The IMH shared these concerns with Sofia and later with her mother and they discussed ways they could help Sofia become more engaged. Sofia’s mother would later report how Sofia played with Maria when no one was watching and how she was implementing all the strategies learned during sessions.

When Maria was 18 months old and not gaining weight, there were concerns that Sofia was not feeding her. Maria was hospitalized and Child Protective Services (CPS) was contacted and an investigation initiated. Since Sofia was a minor and she lived with her family, the investigation involved the whole family. This was a very scary experience for Sofia and her family and a turning point in the treatment when Sofia became more aware of the impact of her quiet approach to service providers. The IMH specialist worked closely with the family and CPS, as well as with hospital staff. She advocated for the family given her concerns about potential bias associated with cultural factors or mother’s young age. The IMH specialist helped Sofia understand not only what the baby was communicating with her behavior but what Sofia communicated with hers. Sofia began participating more actively in the sessions. She would share Maria’s progress, joyfully engaged in play with Maria on a more consistent basis and continued to address all of Maria’s medical needs as she had done since the baby’s discharge from the NICU. After three months when the investigation was completed and it was concluded that poor weight gain could be associated with Maria’s medical condition, the case with CPS was closed.

Overall, throughout the intervention with this family beginning in the FCC, continuing in the NICU, and through sessions in the home, the IMH specialist utilized a Child-Parent Psychotherapy approach with a trauma and developmental lens to understand behavior, support co-regulation, and to strengthen the parent–child relationship and Sofia and Marcos’ parental roles. She also helped link the family to services to reduce stressors impacting their ability to be present for Maria’s needs.

## Discussion

There is a growing recognition regarding the need to provide mental health support within perinatal, neonatal, and postnatal care settings (Hoffman et al., 2020). Establishing an integrated continuum of care infant mental health (IMH) program within a health care system presents unique opportunities in increasing access to behavioral health care during critical periods in the life of a family. The present study outlined a continuum of care IMH program embedded within various medical settings of a large metropolitan children’s hospital. Additionally, descriptive statistics and a case study were provided in order to illustrate the implementation of this unique IMH intervention model.

Embedding IMH services across perinatal and neonatal health care settings increases access to mental health services for families coping with the significant stressors related to birthing and caring for an infant with a medical diagnosis. Additionally, utilizing a continuum of care approach eliminates the stress surrounding having to connect and build rapport with a new provider at each medical juncture. This may include the likelihood of a family having to re-tell their story repeatedly to a new mental health provider in each setting. This is crucial in working with families who are already experiencing heightened stress and may be another way in which to increase access to necessary mental health support.

The population served by the present metropolitan children’s hospital was ethnically/racially diverse and families identified as predominantly Latinx/Hispanic. Prior literature has shown that Latinx communities experience several barriers to accessing behavioral health care including lack of transportation, long waits, inflexible hours, distance between the home and treatment location, lack of health insurance, cost, language, and stigma (Benuto & Leany, 2011). Integrating infant mental health consultation and intervention across a continuum of medical settings aids in reducing these barriers. Prior literature has also demonstrated that although mothers of color are at increased risk for experiencing perinatal and postnatal mood disorders (Bauman et al., 2020), they are less likely to be identified by medical providers for intervention (Kozhimannil et al., 2011). Having IMH specialists embedded within perinatal and neonatal intensive health care units helps to facilitate early identification and referral to appropriate mental health care services.

Our previous study of IMH services in the NICU with the larger sample of referred families explored characteristics that differed between families that engaged in IMH services in the NICU setting versus those that did not (Driver et. al, 2021). That study found language to be the only significant difference, with Spanish-speaking families more likely to engage in services than English-speaking families.

In the present study, we found that families whose children had Medicaid insurance received significantly more IMH service sessions than those with commercial insurance. This is likely due to the fact that grant funding within the outlined program supported families in accessing IMH services in the fetal care center (FCC), neonatal intensive care unit (NICU), and high-risk infant follow-up clinic (HRIF), but only families with Medicaid were eligible for extended outpatient and home-based services through the specialty mental health contract. Although we do not have data regarding mental health services outside our hospital system, IMH clinicians and hospital social workers made efforts to link families with outpatient services through their commercial insurance. However, in our experience it has been difficult to identify providers in the community who accept commercial insurance and have experience treating newborns and providing Child-Parent Psychotherapy or other dyadic interventions. Therefore, linkage to therapists outside the hospital system was primarily for adult therapy for parents experiencing mental health concerns. In order to create a seamless system of care, it is strongly recommended that billing and insurance mechanisms be developed to ensure that services such as those described can be consistently provided in order to meet the mental health needs of all families impacted by medical complexities during perinatal and postnatal periods. Future research is recommended to systematically examine referral pathways and outcomes for families referred to community providers for IMH services.

English-speaking parents were found to be more likely to participate in IMH services within the HRIF setting when compared to Spanish-speaking parents. This is in contrast to the finding from our previous study (Driver et al., 2021) which found that Spanish-speaking families were more likely to engage in IMH services in the NICU setting. HRIF services require that families travel to the hospital location during regular business hours for clinic appointments. It may be that language was a barrier for Spanish speaking families in understanding the nature and importance of the services offered and therefore accessing HRIF services. It is also possible that Spanish-speaking parents experienced additional stressors or worries that may have interfered with them accessing services through HRIF. Future research regarding IMH service provision could explore ways in which to increase engagement in office-based services for Spanish-speaking parents.

Spanish-speaking parents were, on the other hand, more likely to have been seen in their home than English-speaking parents. Three out of the four IMH providers in the study were Latinx and spoke Spanish as their first language. It may be that they made particularly close connections with Spanish-speaking families during prenatal visits and/or NICU hospitalization, as they had opportunities to help parents address the variety of psychosocial needs often present for immigrant families, understand the medical information and communicate with medical providers and were able to help them process their experiences in their primary language. This experience may have then increased the parents’ openness to engagement through home visits due to feeling increased warmth and comfort. The match in language may have also offered opportunities for the IMH providers to help families who were Spanish speaking navigate complex outpatient medical and developmental systems of care, furthering the relationship and trust and support beyond the hospital setting. Another contributing factor could have been that two of the IMH specialists who were Latinx and spoke Spanish had a long history of providing home visitation prior to their involvement in this program. They may have introduced home-visiting services in a manner leading to increased receptivity to home-visiting by families after discharge from the hospital. The case study highlighted that providing high quality IMH services requires utilization of a family-centered and diversity-informed approach. As noted by the diversity-informed IMH tenets (St. John et al., 2012), the IMH specialist provided advocacy for the infant and her family during the CPS investigation in order to combat discrimination. The IMH specialist was also flexible in the ways in which she provided care (i.e., attending medical appointments in order to increase engagement; continuing to reschedule visits when the family had to cancel). This flexibility is particularly important when providing mental health services to families of children with complex medical needs in order to support their engagement. The IMH continuum of care model allows clinicians to provide trauma-informed interventions in a targeted and dose-specific manner based on each family's needs. As demonstrated by the range in number of sessions, there were families that received brief consultations during an acute crisis at a time of a prenatal diagnosis or when hospitalized in the NICU, while others received long-term home-based therapy services. Further, the flexibility of this intervention model lends itself to clinicians being able to easily tailor the therapeutic process to align with each family’s cultural values. For example, the IMH specialist described in the case example was mindful of including the entire extended family unit while providing services to baby Maria and her mother Sofia. Diversity-informed infant mental health tenets highlight the importance of honoring diverse familial structures (St. John et al., 2012). Clinician’s readiness to tailor therapy in a culturally humble manner has been linked to increased engagement and better outcomes for clients (Hook et al., 2013). Therefore, it is important to tailor interventions to align with parents’ cultural and familial values.

This study underscores the value of embedding IMH continuum of care services within health care settings. The present study utilized a case example in order to demonstrate the ways in which an integrated continuum of care IMH program can positively impact the parent-infant bond and maternal and familial well-being. There is a need for future research to further examine the mechanisms through which this IMH intervention model functions across settings when the same providers are embedded in multiple settings providing continuity of care. It would also be important to examine this IMH model when it is implemented with different IMH providers in each setting offering warm hand-offs between providers. Future research should also evaluate the model’s effectiveness in improving long-term outcomes for infants and their families. Additionally, there is a need for integrated continuum of care models to become standard practice within prenatal, neonatal, and perinatal health care settings. These care models should be funded through both commercial and public insurance so that programs without the benefit of philanthropic funding support can make services available to all families. This integrated IMH model would function as a way in which to minimize risk and bolster the parent-infant relationship, which will ultimately promote positive long-term outcomes for infants and their families impacted by medical complexities.

## Data Availability

Due to the nature of this research, participants of this study did not agree for their data to be shared publicly, so supporting data is not available.
